# Mother-to-child transmission of HIV infection and its associated factors in Ethiopia: a systematic review and meta-analysis

**DOI:** 10.1186/s12879-018-3126-5

**Published:** 2018-05-10

**Authors:** Getachew Mullu Kassa

**Affiliations:** College of Health Sciences, Debre Markos University, Debre Markos, Ethiopia

**Keywords:** HIV, PMTCT, MTCT, Prevalence of MTCT of HIV, Vertical HIV transmission, HIV-exposed infant, Systematic review, Meta-analysis, Ethiopia

## Abstract

**Background:**

Mother-to-child transmission (MTCT) is the main mode of HIV transmission in children under 15 years old. This problem is significant in the Sub-Saharan African countries, where more than 80% of children living with HIV are found. Previous studies in Ethiopia present inconsistent and inconclusive findings on the prevalence and associated factors of MTCT of HIV. Therefore, this study was conducted to determine the pooled prevalence of MTCT of HIV and its associated factors in Ethiopia.

**Methods:**

Preferred Reporting Items for Systematic Reviews and Meta-Analyses (PRISMA) guideline was followed. All published studies were retrieved using relevant search terms in MEDLINE, PUBMED, Cochrane Library, EMBASE, Google Scholar, CINAHL, and African Journals Online databases. Joanna Briggs Institute Meta-Analysis of Statistics Assessment and Review Instrument (JBI-MAStARI) was used to critically appraise articles. STATA version 14 software was used to perform the Meta-analysis. The *I*^2^ statistics was used to test heterogeneity and publication bias was assessed using Begg’s and Egger’s tests. Odds ratio (OR) with 95% confidence interval (CI) was presented using forest plots.

**Results:**

A total of nine studies, 3688 mother-baby pairs, were included in this meta-analysis. The pooled prevalence of MTCT of HIV in Ethiopia was 9.93% (95% CI: 7.29, 12.56). The subgroup analysis showed a higher prevalence of MTCT of HIV in Dire Dawa City Administration (15.7%) and lowest in Southern Nations, Nationality and Peoples Region (SNNPR) (4.16%). Associated factors with MTCT of HIV include: mixed feeding, OR = 7.46 (95%CI: 4.71, 11.81), absence of infant ARV prophylaxis, OR = 7.89 (95%CI: 4.32, 14.42), home delivery, OR = 5.08 (95%CI: 2.32, 11.15), and absence of maternal PMTCT intervention, OR = 7.13 (95% CI: 3.31, 15.35).

**Conclusions:**

Almost one in ten HIV exposed infants become HIV positive in Ethiopia. Factors like: mixed feeding, the absence of infant ARV prophylaxis, home delivery and absence of mother’s PMTCT intervention were significantly associated with MTCT of HIV. Therefore, the governmental and non-governmental organizations need to focus on the identified factors and work towards improving the prevention of mother to child transmission of HIV (PMTCT) program.

## Background

Human immunodeficiency virus (HIV) is a virus that weakens the immune system of an individual exposing the body to several opportunistic infections [1]. Although the main mode of HIV transmission is through unprotected sexual intercourse, a significant number of vertical transmission also occurs from mother to child. Mother to child transmission (MTCT) is when HIV is transmitted from the mother to the child during pregnancy, childbirth, or breastfeeding [1–3].

The prevalence of HIV/AIDS has rapidly increased since the 1980s in developing countries. As a result, it has led to several demographic, economic and social consequences [4]. More than 2 million children are living with HIV/AIDS globally, in which more than 80% of them live in sub-Saharan African countries [5]. For example in 2012, 260,000 new pediatric HIV infections occurred, and most of these infections were in Sub-Saharan Africa [6]. The most seriously affected areas in Africa include Southern and Eastern African countries [4]. Hence, the United Nations Program on HIV/AIDS (UNAIDS) set the 90-90-90 target by 2020. The target aims to end the epidemics of HIV by 2030 [7]. The post-2015 HIV priorities plan to dramatically reduce the annual new HIV infection and thereby to save the lives of many peoples [8, 9].

Ethiopia is one of the Eastern African countries with adult HIV prevalence of 1.5% in the population aged 15–49 years old. The prevalence is relatively higher among women than men, with a prevalence of 1.9 and 1.0%, respectively [1]. In 2013, there were more than 160,000 HIV positive children (aged less than 15 years’) in Ethiopia. In addition, the number of orphaned children due to HIV/AIDS were 800, 000. Even though there are a higher number of children with HIV, ART coverage among children was only 12% in the same year [10]. Nonetheless, studies conducted in Ethiopia have shown that the fertility desire among HIV-positive women is still high [11]. For this reason, the Ethiopian Federal Ministry of Health (FMOH) adopted a prevention of mother to child transmission (PMTCT) program aimed at eliminating mother to child transmission of HIV in 2011. PMTCT is a program designed to provide effective interventions during pregnancy, labor and delivery and breastfeeding period for the mother and the baby. The intervention includes the provision of ARV drugs for the mother and the baby and HIV preventive practices. In the absence of such interventions, the risk of MTCT of HIV is 15 to 45% [9]. But through the use of ARV drugs and appropriate preventive mechanisms, the risk can be reduced to less than 5% in under-resourced settings like Ethiopia [2, 3, 9].

Few studies have been conducted on the prevalence of MTCT of HIV and its associated factors in Ethiopia. However, available studies present inconsistent and inconclusive findings in the prevalence of MTCT of HIV and its associated factors. Therefore, this systematic review was conducted to assess the prevalence and factors associated with mother to child transmission of HIV in Ethiopia using available published evidence. The findings of this study will be useful in the design and implementation of proper strategies to reduce the high rate of MTCT of HIV. Likewise, it will be used to monitor the progress of PMTCT program towards Sustainable development goal (SDG-3), target 3.1, 3.2, and 3.3, which aims to ensure healthy lives, end preventable deaths of newborn, and end the epidemics of AIDS by 2030 [12].

## Methods

### Study design and search strategy

This study utilized a systematic review and meta-analysis of published studies. Preferred Reporting Items for Systematic Reviews and Meta-Analyses (PRISMA) guideline was strictly followed during the review and meta-analysis [13]. Published studies were searched in Google Scholar, MEDLINE, PUBMED, Cochrane Library, EMBASE, CINAHL, and African Journals Online databases. This review included all studies which were published from April 2010 to July 7, 2017. The search terms used were “*prevalence of Mother to child transmission of HIV OR prevalence of MTCT of HIV OR factors associated with mother to child transmission of HIV OR HIV-exposed infant OR prevention of mother to child transmission OR PMTCT AND Ethiopia.”*

### Study selection and eligibility criteria

This review included all published studies conducted to assess the rate of HIV transmission from mother to child and its associated factors in Ethiopia. All available studies were included without restricting to a specific study design. The reference list of already selected studies was also screened to retrieve additional articles which can be included in the meta-analysis. Studies published only in the English language were included.

### Quality assessment and data collection

Assessment of articles using their title, abstract, and a full review of the manuscripts was done before the inclusion of articles in the final meta-analysis. Critical appraisal was conducted using Joanna Briggs Institute Meta-Analysis of Statistics Assessment and Review Instrument (JBI-MAStARI) [14]. The criteria included in the instrument includes: random selection of the study sample, clear definition of the criteria for the inclusion of the sample in the study, identification and addressing for confounding factors, use of objective criteria to assess the outcome of interest, reliable measurement of outcome variable and use of appropriate statistical analysis method [14]. The critical appraisal was conducted before the extraction of data. Mean quality score was used to assess the quality of included studies in the meta-analysis. Studies which scored above the mean of the quality score were grouped into the high-quality score, and those below the mean were grouped as the low-quality score.

Data extraction was done using the Joanna Briggs Institute (JBI) tool for prevalence studies [14]. All the necessary information was extracted from the final selected studies using the data extraction tool. The tool contains information on author name and year of publication, study area, study design, study period, time of infant HIV diagnosis, sample size, prevalence of MTCT of HIV, and total number of HIV positive and negative infants by breastfeeding option, infant ARV prophylaxis, place of delivery, and mother’s PMTCT intervention status.

### Outcome of interest

The primary outcome of this review was the prevalence of MTCT of HIV. The Ethiopian national PMTCT guideline recommends diagnosing HIV-exposed infants using the Deoxyribonucleic Acid-Polymerase Chain Reaction (DNA-PCR) virologic tests [9]. The diagnosis is made at six weeks and 18 months using the DNA-PCR test, or rapid antibody test after 6 weeks of breastfeeding cessation. If the child is found to be HIV positive, he/she will be referred to ART clinic for further treatment, care, and support [9]. Additionally, several independent variables were included in this review to determine factors associated with MTCT of HIV. These factors included in this study are: infant breastfeeding option (Exclusive breastfeeding vs mixed feeding), infant ARV prophylaxis (use of prophylaxis vs not using prophylaxis), place of delivery (health facility vs home delivery), and mother’s PMTCT intervention status (received vs not received). The Ethiopian Federal Ministry of Health (FMoH) adopted the Option B+ program for PMTCT in August 2012 [9]. Accordingly, subgroup analysis by the study period in this review was categorized as before 2012 and after 2012, to show the prevalence of MTCT of HIV by the PMTCT program update.

### Heterogeneity and publication bias

The heterogeneity of studies was checked using *I*^*2*^ test statistics and its corresponding *p*-value. A *p*-value less than 0.05 was used to declare heterogeneity. *I*^*2*^ statistics of 25, 50 and 75% was used to declare low, moderate and high heterogeneity, respectively [15]. Egger’s and Begg’s tests were used to assess publication bias, and a *p*-value less than 0.05 were used to declare its statistical significance [16, 17]. The Duval and Tweedie nonparametric trim and fill analysis using the random-effect analysis was conducted for meta-analysis result which showed the presence of publication bias (Egger test, *p* < 0.05) [18].

### Statistical methods and analysis

The extracted data were entered into Microsoft Excel and then was exported to STATA version 14 software for meta-analysis. To calculate the overall pooled prevalence of MTCT of HIV in Ethiopia and its 95% confidence interval (CI), the prevalence rate of MTCT and the standard error (SE) from each study were used. Forest plots were used to present the pooled prevalence of MTCT of HIV with 95% CI. Odds ratio (OR) with 95% CI was also presented in forest plot to show the factors associated with MTCT of HIV. Subgroup analysis was conducted by region of the study, study period and quality score of studies. The meta-analysis was conducted using the random effects model of analysis since it minimizes heterogeneity of the included studies [15].

## Results

### Study selection

**Fig. 1:**
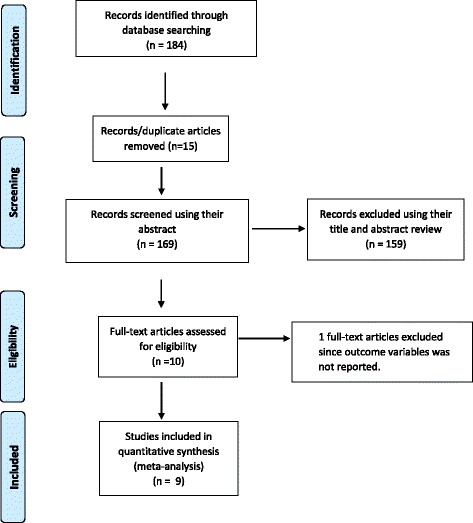
Flow diagram of the studies included in the Meta-analysis

A total of 184 records were retrieved through electronic database searching. Records were screened using their titles, abstracts and through full article review. Accordingly, a total of 159 articles were excluded using their title and abstract review. Ten articles were assessed for eligibility and one article was excluded since the outcome of interest was not reported in the study. Finally, nine articles were included in this meta-analysis (Fig. 1).

### Characteristics of included studies

Eight of the studies included in the final analysis were retrospective cohort studies [19–26] and one study was prospective cohort study [27]. The studies used health facility-based PMTCT data which was collected from 2004 to 2015. All studies included mother-baby pairs who were involved in the PMTCT care in the respective health institutions. Three articles were conducted in Amhara region [20, 21, 26], two in Oromia region [22, 24], two in Addis Ababa, capital city of Ethiopia [19, 27], one study in Southern Nations, Nationality and Peoples Region (SNNPR) [23] and one study was conducted in Dire Dawa City administration [25]. The time of infant HIV diagnosis for all of the included studies was at or after six weeks postpartum. The sample size of included studies ranges from a minimum of 71 mother-baby pairs who had a documented HIV test result in Addis Ababa City [27] to 896 in 10 sub-cities in Addis Ababa city [27]. Overall, a total of 3688 mother-baby pairs were included in this review (Table 1). The quality score of included studies ranges from 6 to 9, with a mean quality score ± SD of 8 ± 0.71.

**Table 1:** Summary characteristics of studies included in the meta-analysis of the prevalence of mother to child transmission of HIV in Ethiopia

Author, year	Study area	Study design	Study period	Sample size	Time of infant HIV diagnosis	Rate of MTCT of HIV
Mirkuzie AH. et al., 2010 [19]	10 sub-cities in Addis Ababa	Retrospective cohort study	February 2004 to August 2009	896	> = 6 weeks	11.8
Koye DN and Zeleke BM., 2013 [20]	Gondar University referral hospital, Amhara	Retrospective cohort study	September 2005 to July 2011	509	> = 6 weeks	10%
Berhan Z. et al., 2014 [21]	South Gondar zone, Amhara region	Retrospective cohort study	January 1 to December 31, 2012	434	> = 6 weeks	10.1%
Derebe G. et al., 2014 [22]	St. Luke Hospital, Woliso town, Oromia	Retrospective cohort study	December 2009 to March 2010	426	> = 6 weeks to 18 months	9.6%
Tadele T. et al., 2014 [23]	Hawassa Referral and Yirgalem General Hospital, SNNPR	Retrospective cohort study	September 2007 to August 2013	485	> = 6 weeks to 18 months, or rapid antibody test after 6 weeks of breast feeding cessation	4.16%
Kumela K. et al., 2015 [24]	Jimma University Specialized Hospital, Oromia	Retrospective cohort study	January 2008 to February 2012	180	> = 6 weeks	15.5%
Wudineh F. and Damtew B., 2016 [25]	Dil Chora Referral Hospital, Dire Dawa City	Retrospective cohort study	July 2005 to July 2013	382	> = 6 weeks to 18 months	15.7%
Moges NA. et al., 2017 [26]	East and West Gojjam Zones, Amhara	Retrospective cohort study	July 2011 to July 2015	305	> = 6 weeks to 24 months follow-up	5.9%
Mirkuzie AH. et al., 2011 [27]	15 health facilities in Addis Ababa	prospective cohort study	January to December 2009	71	At 6 weeks postpartum	8.4%

### Prevalence of MTCT of HIV in Ethiopia

**Fig. 2:**
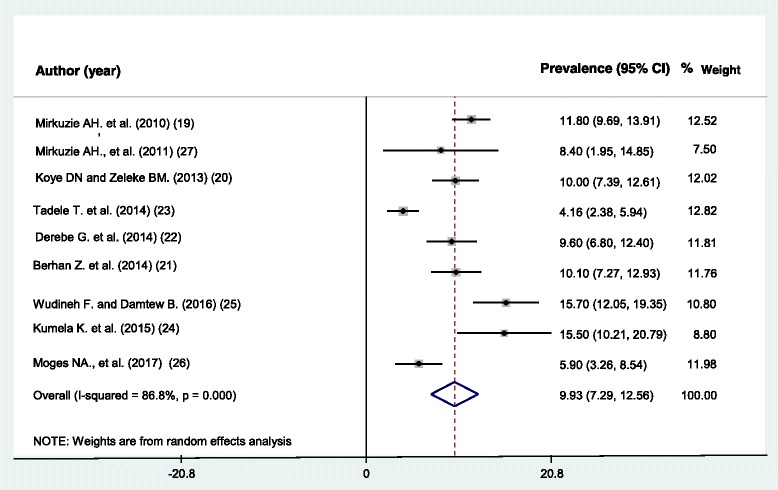
Pooled prevalence of mother to child transmission of HIV in Ethiopia

The prevalence of MTCT of HIV among the included studies ranges from a minimum of 4.16% (95%CI: 2.38, 5.94) in Hawassa University Referral and Yirgalem general hospital, SNNPR [23] to a maximum of 15.7% in DilChora referral hospital, Dire Dawa city administration [25]. A prevalence of 5.9% in East and West Gojjam zones, Amhara region [26], 9.6% in St. Luke Hospital, Woliso town, Oromia region [22], 10% in Gondar University referral hospital, Amhara region [20], and 10.1% in South Gondar zone, Amhara region [21] were also observed. Furthermore, the pooled prevalence of MTCT of HIV in Ethiopia was 9.93% (95% CI; 7.29, 12.56) (Fig. 2). The heterogeneity test showed presence of heterogeneity, *I*^*2*^ = 86.8%, *p*-value = < 0.001. However, non-significant publication bias was detected, *p*-value = 0.143.

### Subgroup analysis

Subgroup analysis was conducted by different study characteristics. The subgroup analysis by region showed the highest prevalence of MTCT of HIV in Dire Dawa City Administration, 15.7% (95%CI: 12.05, 19.35) and the lowest in SNNPR, 4.16% (95%CI:2.38, 5.94), even though only one article were included to each region. The pooled prevalence of MTCT of HIV in Amhara and Oromia regions were 8.65% (95%CI:5.913, 11.38) and 12.11% (95%CI:6.39, 17.82), respectively. The prevalence of MTCT of HIV before the year 2012 was 11.47 (95%CI: 9.46, 13.48) and it reduces to 9.81% (95%CI: 6.72, 12.91) after 2012. The prevalence of MTCT of HIV by quality score of studies was 10.24% in high-quality score studies and 9.55% in low quality score studies (Table 2).

**Table 2:** Subgroup analyses for the prevalence of mother to child transmission of HIV in Ethiopia

Subgroup	Number of included studies	Total sample size	Prevalence (95% CI)	Heterogeneity statistics		
				Tau-Squared	*I* ^*2*^	*p*-value
By region						
Dire Dawa City	1	382	15.70 (12.05, 19.35)	–	–	–
Oromia region	2	606	12.11 (06.39, 17.82)	12.75	73.2	0.053
Addis Ababa City	2	967	11.47 (09.46, 13.48)	0.00	0.00	0.326
Amhara region	3	1248	08.65 (05.91, 11.39)	3.95	67.7	0.045
SNNPR	1	485	04.16 (02.38, 05.94)	–	–	–
By study period						
Before 2012	2	967	11.47 (09.46, 13.48)	0.00	0.00	0.326
After 2012	7	2721	09.81 (06.72, 12.91)	14.89	88.2	< 0.001
By quality score						
Low score	2	665	09.55 (01.00, 20.64)	60.25	93.7	< 0.001
High score	7	3023	10.24 (08.11, 12.38)	5.68	72.2	0.001
Total	9	3688	09..93 (07.29, 12.57)	13.28	86.8	< 0.001

### Mixed feeding and MTCT of HIV

**Fig. 3:**
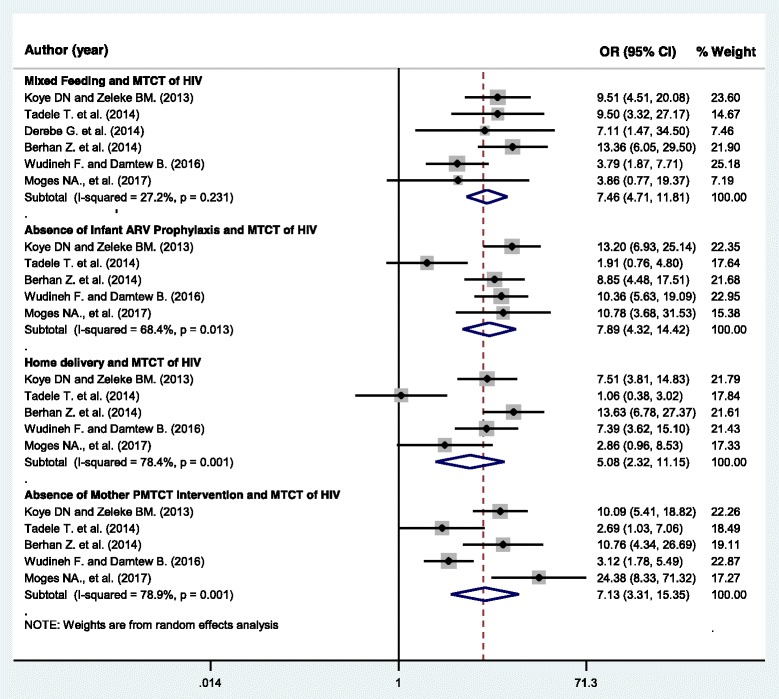
Forest plot displaying the association of selected factors with mother to child transmission of HIV in Ethiopia

Six studies, 2541 mother-baby pairs, were included in this category of meta-analysis [20–23, 25, 26]. Except for one study [26], all other included studies showed the presence of association of mixed feeding with a higher risk of MTCT of HIV. The meta-analysis showed a strong association between mixed feeding and MTCT of HIV, OR = 7.46 (95%CI: 4.71, 11.81). The heterogeneity test showed no statistical evidence of heterogeneity; *I*^*2*^ = 27.2%, *p*-value = 0.231. The Begg’s and Egger’s test for publication bias also showed no statistical evidence of publication bias, *p*-value = 0.573 and *p*-value = 0.892 respectively (Fig. 3).

### Infant ARV prophylaxis and MTCT of HIV

A total of five studies, 2115 mother-baby pairs, were included to assess the association of absence of infant ARV prophylaxis with MTCT of HIV [20, 21, 23, 25, 26]. One study showed a non-significant association between use of infant ARV prophylaxis and mother to child transmission of HIV [23]. Whereas the rest of included articles in this category of the meta-analysis showed a significant association. Accordingly, the pooled meta-analysis showed that infants who didn’t receive ARV prophylaxis were more likely to be HIV-infected than their counterparts, OR = 7.89 (95% CI: 4.32, 14.42). The Begg’s and Egger’s test for publication bias showed no statistical evidence of publication bias, *p*-value = 0.327 and *p*-value = 0.371, respectively. However, statistically significant heterogeneity was observed, *I*^*2*^ = 68.4%, *p*-value = 0.013 (Fig. 3).

### Home delivery and MTCT of HIV

Five studies, 2115 mother-baby pairs, were included in this category of meta-analysis [20, 21, 23, 25, 26]. Three of the included studies [20, 21, 25] showed a significant association between home delivery and a higher risk of HIV transmission from mother to the child. Two studies showed no such association [23, 26]. The pooled meta-analysis showed higher odds of MTCT of HIV among HIV positive women who gave birth at home than women who delivered at health facilities in the presence of skilled birth attendants, OR = 5.08 (95%CI = 2.32, 11.15). Significant heterogeneity (I^2^ = 78.4%, *p*-value = 0.001) was found. Whereas, the Begg’s and Egger’s tests showed no statistical evidence of publication bias, *p*-value = 0.142 and *p*-value = 0.055, respectively (Fig. 3).

### Absence of mother’s PMTCT intervention and MTCT of HIV

Five studies, 2115 mother-baby pairs, were included to determine the association of PMTCT intervention of the mother during pregnancy, or labor and delivery or the postnatal period and risk of MTCT of HIV [20, 21, 23, 25, 26]. The meta-analysis showed that mothers who didn’t use the recommended PMTCT intervention/s during pregnancy/labor and delivery/breastfeeding period are more than seven times more likely to transmit HIV to their child, OR = 7.13 (95%CI: 3.31, 15.36). Heterogeneity test showed evidence of high heterogeneity, *I*^*2*^ = 78.9% and *p*-value = 0.001. However, there was non-significant publication bias, Begg’s test = 0.327 and Egger’s test = 0.460 (Fig. 3).

## Discussion

This meta-analysis was conducted to identify the pooled prevalence of MTCT of HIV and its associated factors in Ethiopia using the available published studies. The review found a higher prevalence of MTCT of HIV in Ethiopia, with the overall pooled prevalence of 9.93% (95%CI: 7.29, 12.56). This progress made is far from what the country had planned to achieve. The post-2015 e-MTCT objective by the nation is to reduce vertical HIV transmission to less than 2% by 2020 [8, 28]. In contrast to the finding of the current study, the previous study conducted in China showed a lower prevalence of MTCT of HIV, 3.9% (95% CI; 3.2, 4.6%) [29]. While a study conducted in South Africa showed a 14% prevalence of MTCT of HIV among infants younger than six weeks and the prevalence was 24% among children aged 3 to 6 months old [30]. A wide variation in the prevalence of MTCT of HIV between developed and developing countries can be attributed to the difference in the sociodemographic, economic, access to Antiretroviral (ARV) drugs, health care coverage and health-seeking behavior of the populations. The poor uptake of PMTCT service in developing countries could also be mentioned as a reason for the higher prevalence of MTCT of HIV. Individual-level factors (poor knowledge of pregnant women, lower level of maternal education, and psychological issues) and community level factors (stigma and fear of disclosure) are the common barriers for poor uptake of PMTCT service [31]. Therefore, addressing individual and community level barriers for poor PMTCT service uptake is important to reduce the high rate of MTCT of HIV in Ethiopia [31].

One of the PMTCT interventions recommended by WHO is the provision of ARV prophylaxis immediately after birth for 6–12 weeks [9]. The duration of infant ARV prophylaxis depends on the ART adherence status of the mother [9]. Nevirapine (NVP) prophylaxis is recommended for six weeks duration for infants who are breastfeeding, and for 4 to 6 weeks of NVP prophylaxis for infants who are not breastfeeding [6]. The meta-analysis of this review showed that infants who didn’t receive ARV prophylaxis at or after birth are more than seven times more likely to be HIV-infected than an infant who received ARV prophylaxis. Several studies also mentioned the importance of infant ARV prophylaxis in preventing mother to child transmission of HIV [6, 32]. The ARV prophylaxis given to the infant serves as pre or post-exposure prophylaxis to HIV and it can protect the infant against the HIV especially during breastfeeding [6, 32, 33].

This review also found that infants who were on mixed feeding before the age of six months were more than seven times more likely to be HIV positive than infants who were on exclusive breastfeeding. This could be because mixed feeding is associated with gastrointestinal ulceration secondary to diarrheal disease. As a result, the virus can quickly enter the infant’s bloodstream through the ulcerated gastrointestinal tissue [6, 9, 34, 35]. In light of this, WHO and the current Ethiopian national PMTCT guideline recommends the use of safe infant feeding options which include: exclusive breastfeeding for the first six months and initiating complementary foods at six months to 12 months of infant’s life and to avoid mixed feeding before six months of infant’s life [9, 34]. A study conducted in Nigeria also showed a higher risk of HIV infection among HIV exposed infants who were on mixed feeding [36].

The use of skilled delivery attendance at birth can reduce the risk of morbidity and mortality for both the mother and the child [1, 37]. The current review also found that HIV positive mothers who deliver at home were five times more likely to have HIV positive child than HIV positive women who attended skilled birth attendant at a health facility. This could be due to the lack of PMTCT interventions during and immediately after labor and delivery for mothers who gave birth at home. Moreover, interventions available at health facilities include the use of standard infection prevention practices, use of partograph to follow the progress of labor, use of ARV prophylaxis, and safe delivery practices [9, 34]. A study conducted in western Europe also found that delivery of the baby by elective cesarean section can prevent mother to child transmission of HIV [38]. A similar finding was also observed in a study conducted in Italy [39].

The presence of PMTCT intervention during pregnancy, labor and delivery, and breastfeeding period is essential in the reduction of the HIV-positive child [9]. The findings of this review showed that HIV positive women with no PMTCT intervention were more than seven times more likely to have HIV positive child. Likewise, without any maternal and/or child PMTCT intervention, 20 to 45% of infants will be HIV-infected [2, 3, 9]. This could be due to the benefits of ARV drugs in reducing maternal viral load, and thereby reducing the risk of HIV transmission from mother to the child [9, 28]. WHO report also showed that ARV prophylaxis to a woman and her infant could reduce the risk of mother to child transmission to less than 2% [40].

This review strictly followed the PRISMA guideline during the review and meta-analysis process. All eligible studies on MTCT of HIV were included. However, only studies published in the English language were included. Unpublished research works or government reports were not included in this review. Furthermore, this review included only a few variables associated with MTCT of HIV in Ethiopia, because of the limited number of studies. However, previous studies mentioned older maternal age [26], HIV positive mothers who didn’t follow antenatal care [22], late enrolment to HIV exposed infant follow up clinic [20], short duration of ART regimen [21, 24], low maternal CD4 count (less than 350 cells/cubic mm) at baseline [24], mothers on WHO clinical stage 3 and 4 [22], and low infant birth weight (less than 2500 g) [24] as additional factors associated with MTCT of HIV. The outcome variable may also be affected by other confounding variables not mentioned in this review. Therefore, further nationwide study to assess personal, health service factors and policy-related reasons for a higher rate of MTCT of HIV in Ethiopia is recommended.

## Conclusions

The country has made good progress in reducing the rate of mother to child transmission of HIV. However, the rate of reduction is slow to achieve the elimination of mother to child transmission of HIV goal by 2020. Moreover, this review showed that almost one in every ten HIV-exposed infants become HIV positive. The prevalence of MTCT of HIV varies across different regions of the country. Higher risk of MTCT of HIV was observed among HIV exposed infants who didn’t take ARV prophylaxis, who were on mixed feeding before six months of age, who were delivered at home, and whose mother was not on PMTCT intervention. This calls the Ministry of Health and other concerned partners to focus on the identified factors and work towards improving the PMTCT program. The use of ARV prophylaxis by the mother during pregnancy, and breastfeeding period, and use of infant ARV prophylaxis should be strengthened. HIV testing and counseling programs for women and their partner should be enhanced at antenatal, labor and delivery, and postnatal settings. Also, institutional and community-based comprehensive health education programs on the importance of skilled birth attendance, postpartum care and maternal and infant PMTCT interventions is essential. Further studies to identify the national prevalence and possible additional associated factors especially in regions with higher prevalence of MTCT of HIV are needed.

## Abbreviations

ART: Antiretroviral therapy
ARV: Antiretroviral
CI: Confidence interval
DNA: Deoxyribonucleic acid
EDHS: Ethiopian Demographic and Health Survey
e-MTCT: Elimination of mother-to-child transmission of HIV
FMOH: Federal Ministry of Health
HIV: Human immunodeficiency virus
MTCT: Mother-to-child transmission
OR: Odds ratio
PCR: Polymerase chain reaction
PMTCT: Prevention of mother-to-child transmission
SNNPR: Southern Nations, Nationality and Peoples Region
WHO: World Health Organization
